# Adsorption of Ferritin at Nanofaceted Al_2_O_3_ Surfaces

**DOI:** 10.3390/ijms241612808

**Published:** 2023-08-15

**Authors:** Bhanu K. Pothineni, Sabrina Kollmann, Xinyang Li, Guido Grundmeier, Denise J. Erb, Adrian Keller

**Affiliations:** 1 Technical and Macromolecular Chemistry, Paderborn University, Warburger Str. 100, 33098 Paderborn, Germany; bhanu.kiran.pothineni@uni-paderborn.de (B.K.P.); sschwid2@mail.uni-paderborn.de (S.K.); xinyangl@mail.uni-paderborn.de (X.L.); guido.grundmeier@uni-paderborn.de (G.G.); 2 Ion Beam Center, Institute of Ion Beam Physics and Materials Research, Helmholtz-Zentrum Dresden-Rossendorf, 01328 Dresden, Germany

**Keywords:** biointerfaces, nanopatterning, self-organization, sapphire

## Abstract

The influence of nanoscale surface topography on protein adsorption is highly important for numerous applications in medicine and technology. Herein, ferritin adsorption at flat and nanofaceted, single-crystalline Al_2_O_3_ surfaces is investigated using atomic force microscopy and X-ray photoelectron spectroscopy. The nanofaceted surfaces are generated by the thermal annealing of Al_2_O_3_ wafers at temperatures above 1000 °C, which leads to the formation of faceted saw-tooth-like surface topographies with periodicities of about 160 nm and amplitudes of about 15 nm. Ferritin adsorption at these nanofaceted surfaces is notably suppressed compared to the flat surface at a concentration of 10 mg/mL, which is attributed to lower adsorption affinities of the newly formed facets. Consequently, adsorption is restricted mostly to the pattern grooves, where the proteins can maximize their contact area with the surface. However, this effect depends on the protein concentration, with an inverse trend being observed at 30 mg/mL. Furthermore, different ferritin adsorption behavior is observed at topographically similar nanofacet patterns fabricated at different annealing temperatures and attributed to different step and kink densities. These results demonstrate that while protein adsorption at solid surfaces can be notably affected by nanofacet patterns, fine-tuning protein adsorption in this way requires the precise control of facet properties.

## 1. Introduction

The interaction of proteins with solid surfaces plays an important role in various fields of modern medicine. The adsorption of numerous proteins from blood and other physiological media at implant surfaces critically affects the response of the surrounding tissue and thus may decide over implant integration or failure [1]. Protein adsorption, however, is also of relevance for other medical devices beyond implants. This, in particular, concerns any equipment in direct contact with blood as the adsorption of plasma proteins may lead to blood clot formation and thus have severe consequences for the device or the patient [2]. Promoting the specific adsorption of target proteins while simultaneously suppressing the nonspecific adsorption of non-targeted species furthermore is an important prerequisite in biomarker detection and disease diagnostics [3]. Finally, protein adsorption is also the initial step in the adhesion of pathogenic bacteria [4,5] and viruses [6,7] to everyday surfaces and, thus, may influence the spread of infectious diseases.

However, protein adsorption at solid-liquid interfaces is not only an important but also a highly complex phenomenon and influenced by a large variety of environmental parameters [8,9,10]. Important solution parameters in this regard are pH and ionic strength, the presence of cosolutes, temperature, and the concentration and species of the protein. Protein properties known to influence adsorption are, in particular, the protein’s charge and hydrophobicity, its glycosylation state, and its stability under the given environmental conditions. Consequently, different proteins usually show very different adsorption behavior under otherwise identical conditions. The same also goes for the solid surface with the adsorption of a given protein usually proceeding rather differently at different surfaces, resulting in altered adsorption kinetics, differences in adsorbed protein mass at equilibrium, and different conformations of the adsorbed proteins. The surface properties responsible for these differences are mostly the surface chemistry, i.e., surface charge and hydrophobicity, propensity to participate in van der Waals interactions and H bonding, and hydration behavior. Furthermore, it is now understood that the nanoscale topography of the surface can also have a pronounced effect on protein adsorption [11,12,13,14].

In our previous works, we have investigated the adsorption of various globular proteins with widely different properties, i.e., myoglobin, serum albumin, and thyroglobulin, at nanopatterned and nanorough titanium oxide and silicon oxide surfaces [15,16]. We found that protein adsorption in general is rather sensitive toward the presence of topographic surface features with vertical dimensions well below 10 nm. Depending on the protein in question, the surface material, and the exact surface morphology, surface nanopatterning can both promote and retard protein adsorption. Furthermore, we observed that surface nanopatterning in this particular size range can also retard the aggregation of amyloidogenic peptides at silicon oxide surfaces [17]. Surface nanopatterning thus represents a promising route to fine-tuning protein-surface interactions.

In the present paper, we extend our previous studies and investigate the adsorption of the protein ferritin at nanofaceted Al_2_O_3_ surfaces. Al_2_O_3_ is an important biomaterial especially for dental applications [18,19], and many research efforts aim at improving its biocompatibility via various nanopatterning strategies [20,21,22]. Ferritin is found in almost all organisms and cell types [23]. Its main physiological roles are in iron storage and transport, but it is also involved in immunity, inflammation, and angiogenesis, amongst others [24]. For decades, serum ferritin has been used as a biomarker of iron-storage-related diseases such as iron deficiency anemia [24]. It further has the potential to serve as a prognostic biomarker also for other diseases such as cancer [25] and COVID-19 [26]. In addition, ferritin is also used in materials science and nanotechnology—for instance, in the synthesis of various inorganic nanoparticles and quantum dots, as a catalyst for carbon nanotube growth, as an etch mask in surface patterning, and as a charge storage node in memory devices [27]. Controlling the interaction of ferritin with solid surfaces, thus, is an important issue in medicine and technology.

Ferritin adsorption at single-crystalline Al_2_O_3_($10\bar{1}0$) substrates was studied using atomic force microscopy (AFM) and X-ray photoelectron spectroscopy (XPS). Thermal annealing of these substrates at temperatures between 1300 and 1500 °C led to the formation of faceted saw-tooth-like surface topographies with periodicities of about 160 nm and amplitudes of about 15 nm [28,29]. Even though the dimensions of the so-generated patterns are larger than the diameter of the protein, ferritin adsorption at these nanofaceted surfaces is notably altered compared to flat references surfaces. At low protein concentrations, our results suggest that the formed facet surfaces of R-plane, i.e., ($1\bar{1}02)$ orientation, and S-plane, i.e., approximately ($10\bar{1}1)$ orientation, respectively, have a lower affinity for ferritin than the original Al_2_O_3_($10\bar{1}0$), i.e., M-plane, surface, so that sizeable ferritin adsorption is limited to the groove valleys, where the contact area between protein and surface is maximized. At higher protein concentrations, however, ferritin adsorption at the nanofaceted surfaces is enhanced compared to the flat surface. We further demonstrate that topographically similar nanofacet patterns prepared under different conditions can display notably different behaviors in contact with the protein.

## 2. Results

### 2.1. Substrate Surface Characterization

**Figure 1:**
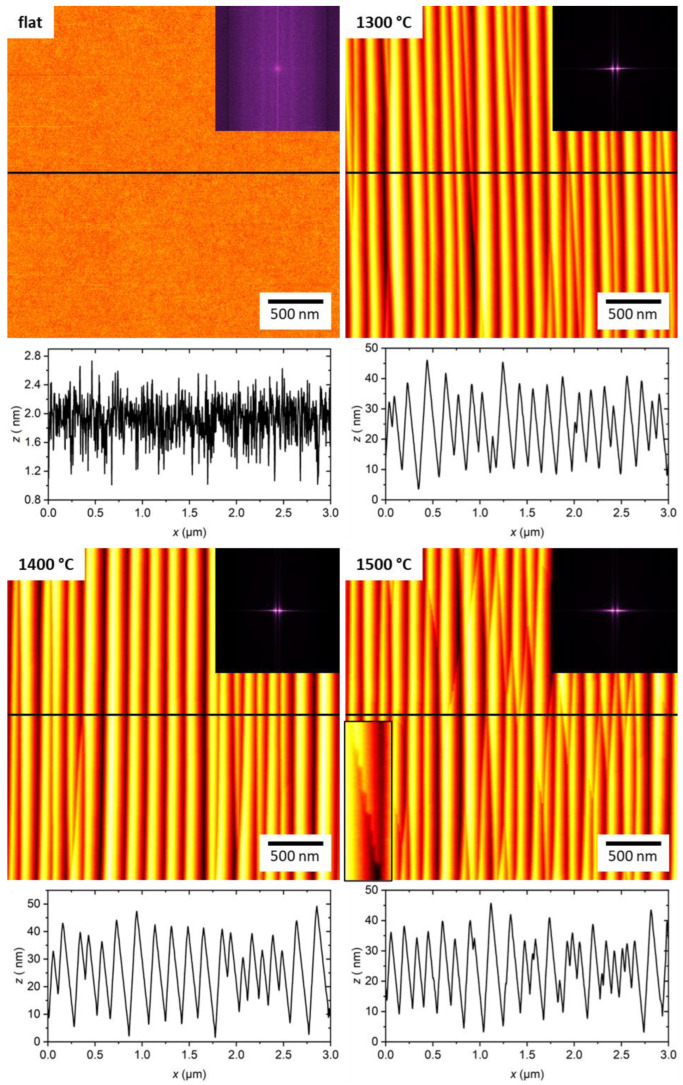
AFM images of the different Al_2_O_3_ substrates before ferritin adsorption. Below each image, a height profile is shown that was taken along the horizontal line indicated in the AFM image. The maximum of the height scales is 4 nm for the flat and 50 nm for the nanofaceted surfaces, respectively. Upper right insets show the 2D Fast Fourier Transforms (FFTs) of the AFM images. The lower left inset in the image of the 1500 °C surface shows a zoom (117 × 401 nm^2^) of an angled step crossing a facet.

AFM images of the untreated and the annealed Al_2_O_3_ substrates are shown in Figure 1. As one would expect for a single crystal, the untreated substrate has a very flat surface morphology without any pronounced textures (see the Fast Fourier Transform, FFT, in the inset). Annealing at temperatures above 1000 °C, however, results in the appearance of rather ordered ripple patterns with an asymmetric sawtooth profile and a periodicity and length of about 150 nm and several microns, respectively. Thus, the surface topography displays a strong anisotropy at the corresponding length scales, as can be seen in the FFT. Varying the annealing temperature between 1300 and 1500 °C does not lead to any pronounced differences in the pattern periodicity. All surfaces show pattern defects in the form of edges that cross the facets at approximately ±10° with respect to the $\left[1\bar{2}10\right]$ direction of the main facet edges (see also Figures S2–S4). However, the 1500 °C substrate shows more such defects, and close inspection of the AFM images also reveals that many of these defects are composed of a series of kinks (see lower left inset in the corresponding AFM image in Figure 1).

The major morphological parameters of the untreated and the annealed Al_2_O_3_ substrates, i.e., the root-mean-square (RMS) surface roughness *S*_q_, the roughness factor *r*, which is the ratio of the real 3D surface area and the projected 2D surface area, and the periodicity λ and peak-to-peak amplitude *A*_p2p_ of the patterns, were determined from the AFM images and are listed in Table 1. Nanopattern formation leads to a drastic increase in *S*_q_ from about 0.3 nm to about 8.4 nm for 1300 °C annealing to about 10.1 nm for 1400 °C annealing. At 1500 °C, however, *S*_q_ drops again to about 8.0 nm. Similar trends are also observed for the pattern periodicity and amplitude. For the surface treated at 1300 °C, λ~162 nm and *A*_p2p_~15 nm are determined. For higher temperature annealing at 1400 °C, these values increase to λ~178 nm and *A*_p2p_~18 nm, whereas a reduction to λ~146 nm and *A*_p2p_~15 nm is observed at 1500 °C, respectively. However, it should be noted at this point that both quantities are somewhat ill-defined for such faceted patterns. This is already indicated by the 2D FFTs in Figure 1, which show streaks instead of well-defined correlation peaks. Therefore, these quantities should be considered as rough measures of the exact pattern dimensions. The roughness factor on the other hand is a more reliable quantity. While *r* = 1.01 for the untreated surface indicates a very flat topography, thermal annealing leads to an increase of *r* to 1.08 to 1.09 and thus to considerably larger surface areas for all three treated temperatures. Therefore, as long as the affinity of a single ferritin molecule for the Al_2_O_3_ surface is not altered by the change in surface topography, the increase in effective surface area should result in a larger amount of adsorbed protein under otherwise identical conditions.

Finally, the chemical composition of the different Al_2_O_3_ surfaces was analyzed through XPS. All four substrate surfaces were found to be composed only of aluminum and oxygen with an additional 8 to 16 at% of adventitious carbon (see Table 2). Notably, the aluminum content is almost identical for each substrate, which demonstrates that the thermal treatment does not alter the chemical composition of the Al_2_O_3_ surface in any way.

### 2.2. Ferritin Adsorption

**Figure 2:**
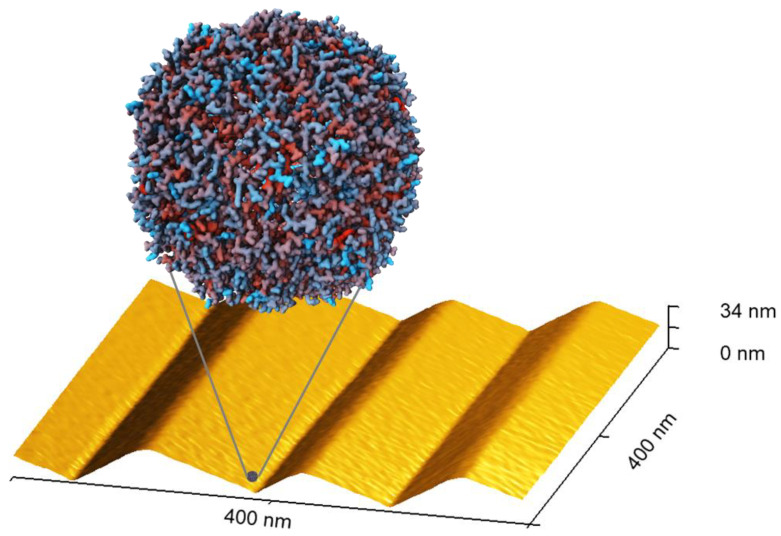
Molecular structure of ferritin taken from the RCSB PDB (rcsb.org), PDB ID 2W0O [30] and size comparison with the topography of a representative nanofaceted Al_2_O_3_ surface. The image of the protein surface was created using Mol* Viewer [32], and the colors indicate the hydrophobicity of the residues from blue (lowest) to red (highest). The 3D representation of the nanofacet pattern is a zoomed AFM image of a substrate annealed at 1400 °C.

**Figure 3:**
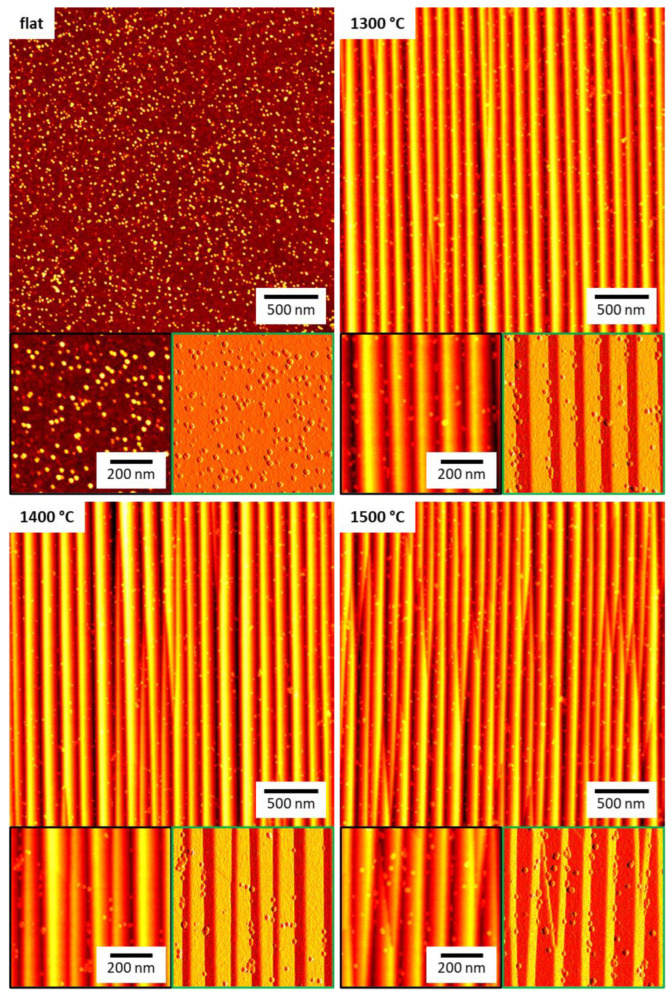
Overview AFM images of the different Al_2_O_3_ substrate surfaces after ferritin adsorption at 10 mg/mL. The maximum of the height scales is 12 nm for the flat and 50 nm for the nanofaceted surfaces, respectively. Below each overview image, zooms are shown with the topography channel on the left (black) and the corresponding peak force error channel on the right (green).

The flat and the nanofaceted Al_2_O_3_ surfaces were exposed to 10 mg/mL horse spleen ferritin in phosphate-buffered saline (PBS) at room temperature for five hours. This time was selected to achieve saturation of the surface coverage. The surfaces were then imaged by AFM in the dry state. Ferritin is a protein cage with 10 to 12 nm outer diameter that encloses a ferrihydrite-like nanoparticle about 5 to 8 nm in diameter (see Figure 2) [23,27,30]. Therefore, individual adsorbed ferritin particles can be easily resolved by AFM, even though they are considerably smaller than dimensions of the nanofacet patterns (see Figure 2). As can be seen in Figure 3, the flat Al_2_O_3_ surface shows a high surface coverage with protein particles. Close inspection of the corresponding zoomed topography image (black) reveals two species of protein particles. The larger species has heights between 8 and 10 nm, while the smaller species has heights of only 2 to 4 nm. Therefore, we assign the former species to iron-filled ferritin and the latter to apoferritin that lacks a nanoparticle core and thus collapsed during adsorption and drying [31].

For the nanofaceted surfaces, the AFM images in Figure 3 seem to reveal an overall reduced surface coverage compared to the flat surface despite the increased effective surface area as quantified by the roughness factor *r* (see Table 1). However, because of the comparatively large amplitudes of the nanopatterns, individual ferritin particles are more difficult to identify in the topography images. Therefore, we also provide zoomed peak force error images in Figure 3, which do not suffer as much from the large differences in surface height. Indeed, for all three samples, these images show rather low amounts of ferritin adsorbed on the facet planes. Furthermore, these images also reveal preferential ferritin adsorption at the bottom of the grooves, i.e., right where the two facet planes meet. This suggests that the two facets formed during thermal annealing have lower affinities for ferritin than the original Al_2_O_3_($10\bar{1}0$) surface. In the grooves, however, ferritin molecules can occupy binding sites that facilitate contact with each of the two facet planes, which maximizes their contact area with the surface and results in higher binding affinity.

**Figure 4:**
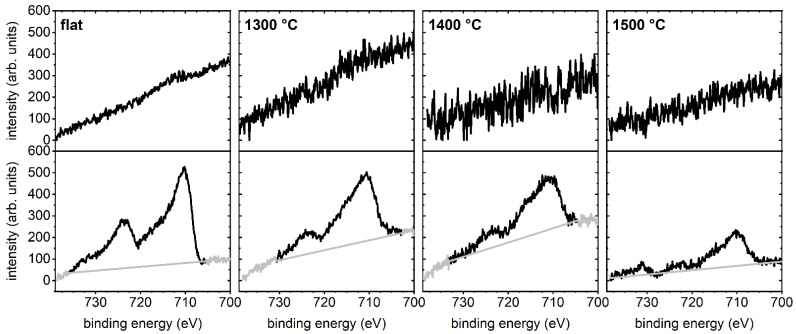
High-resolution XPS data showing the Fe 2p region of the different Al_2_O_3_ surfaces before (top) and after (bottom) ferritin adsorption at 10 mg/mL. The background used for quantification is indicated in grey. The spectra were shifted vertically for clarity (see also Figure S17).

In order to quantitatively assess differences in the adsorbed amount of ferritin at the flat and nanofaceted Al_2_O_3_ surfaces, we turned to XPS. Each ferritin molecule can store up to 4500 Fe^3+^ ions, rendering the surface concentration of Fe a convenient measure of the amount of adsorbed proteins [33,34,35]. Figure 4 shows high-resolution spectra of the Fe 2p region for all four surfaces before and after adsorption of ferritin at 10 mg/mL. In all cases, sizable Fe signals can be observed only after ferritin adsorption. In agreement with the AFM data, the highest intensity of the Fe 2p peaks is observed for the flat surface. The surfaces annealed at 1300 and 1400 °C show lower and rather similar peak intensities. For the 1500 °C surface, the Fe 2p peak intensity is further reduced.

**Figure 5:**
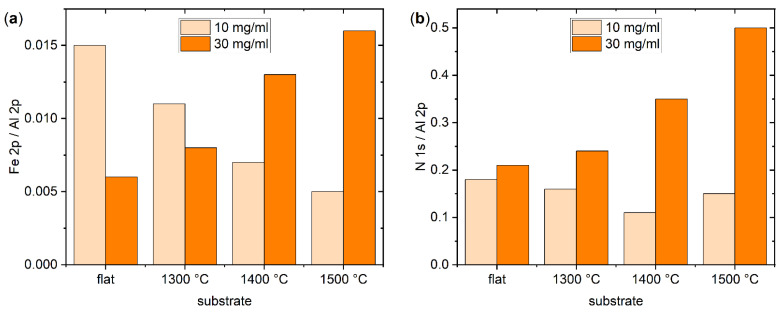
(**a**) Fe 2p/Al 2p ratio (at%/at%) as measured by high resolution XPS and (**b**) N 1s/Al 2p ratio (at%/at%) as determined from the XPS survey spectra for the different Al_2_O_3_ substrate surfaces after ferritin adsorption at 10 mg/mL and 30 mg/mL, respectively.

These qualitative observations are further substantiated in Figure 5a, which quantitatively compares the Fe 2p/Al 2p ratio of the different Al_2_O_3_ surfaces determined from the high-resolution XPS data in Figure 4 and Figure S16. For the flat surface, ferritin adsorption is accompanied by an Fe 2p/Al 2p ratio of 0.015. This value decreases to 0.011 and 0.007 for the nanofaceted surface annealed at 1300 and 1400 °C, respectively. Annealing at 1500 °C leads to a further reduction to 0.005. This decrease in ferritin surface coverage with increasing annealing temperature is rather surprising considering the chemical and topographic surface properties listed in Table 1 and Table 2. While all three surfaces have essentially the same chemical composition, their surface topographies vary to some extent. However, as can be seen in Table 1, the 1400 °C substrate exhibits the largest and the 1500 °C substrate the smallest pattern periodicity λ and peak-2-peak amplitude *A*_p2p_. The λ value of the 1300 °C substrate lies almost right in the middle between those of the other two substrates, whereas its *A*_p2p_ value is almost identical to that of the 1500 °C substrate.

**Figure 6:**
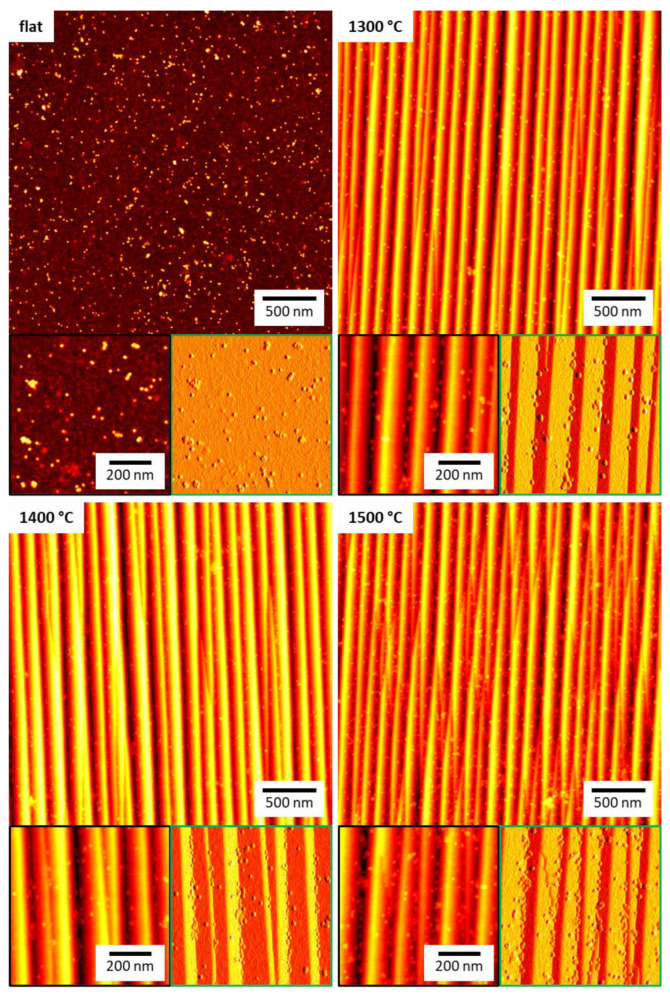
Overview AFM images of the different Al_2_O_3_ substrate surfaces after ferritin adsorption at 30 mg/mL. The maximum of the height scales is 12 nm for the flat and 50 nm for the nanofaceted surfaces, respectively. Below each overview image, zooms are shown with the topography channel on the left (black) and the corresponding peak force error channel on the right (green).

In order to test whether this peculiar behavior persists also at higher ferritin concentrations, the above experiments were repeated at 30 mg/mL. Surprisingly, the corresponding AFM image in Figure 6 reveals a reduced surface coverage of the flat surface (see also Figures S5 and S9). This is a rather unexpected behavior since even for denaturing proteins, a higher concentration should result in a larger surface coverage [9]. However, close inspection of the AFM images reveals a large number of smaller structures in the background that cover the surface almost completely. We believe that these structures are adsorbed apoferritin, which apparently has a higher affinity for the flat Al_2_O_3_ surface than ferritin, presumably because it can more easily deform [31] and thereby maximize its contact area with the surface. The increased adsorption of apoferritin then blocks possible adsorption sites for ferritin, resulting in lower ferritin surface coverage. This interpretation was further substantiated by XPS. As can be seen in Figure 5a, the Fe 2p/Al 2p ratio of the flat substrate surface decreases from 0.015 at 10 mg/mL to 0.006 at 30 mg/mL. At the same time, however, the N 1s/Al 2p ratio increases from about 0.18 to 0.21, indicating increased adsorption of protein species.

For the nanofaceted surface annealed at 1300 °C, the AFM images in Figure 6 do not show such a strong apoferritin background, and the ferritin surface coverage appears comparable to the that of the lower concentration of 10 mg/mL, both of which are supported by the XPS data in Figure 5. Here, a smaller decrease in the Fe 2p/Al 2p ratio from 0.011 to 0.008 is observed, while the increase in the N 1s/Al 2p ratio is slightly larger than that of the flat surface. A clear increase in the Fe 2p/Al 2p ratio is observed for the 1400 °C surface, and the N 1s/Al 2p ratio shows a stronger increase than for the other two surfaces, i.e., from 0.11 to 0.35. Nevertheless, the corresponding AFM images in Figure 6 and Figure S12 reveal that ferritin adsorption is still predominantly restricted to the grooves, while the facet planes are mostly free of adsorbed proteins. For the 1500 °C substrate, on the other hand, an increase in surface coverage is clearly visible in Figure 6. Consequently, the XPS data in Figure 5a show a drastic increase in the Fe 2p/Al 2p ratio from 0.005 at 10 mg/mL to 0.016 at 30 mg/mL. This increase is accompanied by a similarly dramatic increase in the N 1s/Al 2p ratio from 0.15 to 0.50 (Figure 5b). In sum, these data demonstrate that the nanofaceted surfaces not only suppress but modulate ferritin and apoferritin adsorption in a nontrivial way.

## 3. Discussion

The data presented above suggest that adsorption of ferritin at low concentrations is suppressed at all three nanofaceted Al_2_O_3_ surfaces compared to the flat surface. At high ferritin concentrations, however, ferritin adsorption is increased on the nanofaceted surfaces. The lateral dimensions of the faceted nanopatterns are much larger than the protein, and protein adsorption at the flat facet planes is notably reduced for all three nanofaceted surfaces and both concentrations. Therefore, we assume that the facets generated by high-temperature annealing have lower affinities for the ferritin protein than the original Al_2_O_3_($10\bar{1}0$) surface. This is in agreement with previous observations of crystal plane-dependent adsorption on different Al_2_O_3_ surfaces.

Yamazaki et al. investigated the adsorption of different proteins including ferritin on cross-stepped Al_2_O_3_(0001) surfaces [36]. These were generated by thermal annealing of miscut crystals, which resulted in the simultaneous appearance and the overlay of steps in different crystallographic directions, i.e., [$1\bar{1}00$] and [$1\bar{2}10$]. The enclosed crystal planes underwent phase separation that resulted in the formation of a hydrophobic domain within each hydrophilic domain [37]. For ferritin at neutral pH, this hydrophilic-hydrophobic pattern resulted in selective adsorption at the hydrophobic domains and suppressed adsorption at the hydrophilic ones [36]. In the current experiments, however, the situation is more complex, as the formed facets differ in their crystallographic orientation from the original Al_2_O_3_($10\bar{1}0$) surface and ferritin adsorption appears to be suppressed at both facets.

High-temperature annealing of the Al_2_O_3_($10\bar{1}0$), i.e., M-plane, surface leads to the formation of a hill-and-valley morphology composed of two different facets with ($1\bar{1}02$), i.e., R-plane, and roughly ($10\bar{1}1$), i.e., S-plane, orientation [28,29,38]. This self-organized patterning is the result of surface energy minimization: the initial M-plane surface has a comparatively high surface energy density, but surface energy minimization via crystal surface reconstruction is only enabled by increased diffusivity at high temperatures. While maintaining its crystallinity, the surface then reconstructs into alternating facets of R-plane and S-plane orientation, which intersect at edges along the $\left[1\bar{2}10\right]$ direction. The resulting rippled morphology has a larger surface area but still reduces the total surface energy due to the lower surface energy densities of the R- and S-planes. Mesoscopically, the surface reconstruction starts with the nucleation of isolated ripples at asperities of the initial surface, which in turn provoke the formation of further adjacent ripples. These ripple groups grow by elongation and coarsening of the ripples until they coalesce. The coalescence leaves pattern defects where the ripple edges of two ripple groups do not align.

The M-plane and the R-plane have similar isoelectric points around pH 5.9 and similar zeta potentials at pH 7.4 [39]. Ferritin, on the other hand, has an isoelectric point between pH 4.6 and 5.0 [40]. Therefore, at pH 7.4, the M-plane and the R-plane both will be negatively charged, as will the protein. Different studies have further shown that the adsorption of various organic species at the R-plane surface is only slightly less efficient than at C-plane Al_2_O_3_(0001) [41,42,43], even though the C-plane has a lower isoelectric point and zeta potential [39]. It thus appears unlikely that electrostatic interactions with the R-plane facets are responsible for the observed differences in ferritin adsorption.

Less is known about the physicochemical properties of the S-plane, which develops kinks on the pattern defects during 1500 °C annealing that are identifiable in Figure 1. The S-plane is a complex surface [28,29,38], which does not correspond exactly to any crystal plane of Al_2_O_3_ and may therefore decay further into steps with heights of several lattice unit cells parallel to the facet edges along $\left[1\bar{2}10\right]$. The higher number of pattern defects on this substrate surface possibly results from a higher nucleation density of ripples on the initial ($10\bar{1}0)$ surface at the beginning of the thermal treatment. The fact that the defects are composed of large kinks visible in AFM may be a consequence of the increased diffusivity at higher annealing temperatures. An energetically favorable state with few large kinks is then achieved in shorter time than at lower annealing temperatures.

Close inspection of the corresponding AFM images in Figure 6 and Figure S12 furthermore reveals that the S-plane facets also exhibit a larger number of adsorbed ferritin molecules than the R-plane facets. Given the higher diffusivity at 1500 °C as indicated by the formation of kinks on the pattern defects, the decomposition of the S-plane into steps may also have proceeded further than in the other samples, albeit not yet being observable in AFM. This step formation would result in an increase in the local surface roughness, which is known to affect protein adsorption in a complex manner [16]. Therefore, we assume that the particular properties of the S-plane are responsible for the notably different behavior of the 1500 °C surface, which shows lower and higher ferritin coverage than the other nanofaceted surfaces at 10 and 30 mg/mL, respectively.

In summary, our results show that protein adsorption at solid surfaces can be notably affected by nanofaceting. For the comparatively large pattern dimensions investigated in this work, i.e., about 160 nm periodicity and about 15 nm amplitude, surface topography effects do not seem to play a dominant role. Rather, it appears that protein adsorption is governed by the affinities of the formed facets for the protein in question. For the nanofaceted Al_2_O_3_ surfaces employed in this study, a suppression of protein adsorption is observed at the facet planes at low protein concentrations. Under this condition, notable protein adsorption is limited to the grooves of the nanopatterns, where the reduced affinity for the protein is counteracted by an increased contact area. At higher protein concentrations, however, ferritin adsorption occurs also on the facet surfaces, resulting in higher surface coverage than for the flat surface. As a side effect of this variation of protein adsorption at different facets, we also observed that variations in fabrication conditions, i.e., annealing temperature, may result in rather different protein adsorption behaviors, even though the generated surfaces have rather similar topographies.

## 4. Materials and Methods

### 4.1. Fabrication of Nanofaceted Al_2_O_3_ Surfaces

Single-side polished single-crystalline wafers of Al_2_O_3_($10\bar{1}0$) with a size of 10 × 10 mm² (CrysTec GmbH Kristalltechnologie, Berlin, Germany) were cleaned by wiping with ethanol and annealed in air using a tube furnace (Stroehlein CTF 16/50, Carbolite Furnaces, Neuhausen, Germany). Substrates were annealed at 1300 °C, 1400 °C, and 1500 °C, respectively, with an identical heating rate of 300 K/h, annealing duration of 720 min, and cooling rate of 250 K/h. The crystal surface reconstruction enabled by enhanced diffusivity at these high temperatures results in the spontaneous formation of an anisotropic pattern of nanoscale facts with a saw-tooth profile that covers the entire substrate surface.

### 4.2. Ferritin Adsorption

The flat and nanofaceted Al_2_O_3_ substrates were cleaned through incubation in 2% Hellmanex solution (Hellma GmbH & Co. KG, Müllheim, Germany) for 1 h at room temperature, washing with HPLC-grade water (Carl Roth GmbH, Karlsruhe, Germany), and drying in a stream of argon. Finally, the substrates were treated for 30 s with an oxygen plasma (diener Zepto, Diener electronic, Ebhausen, Germany) to remove any remaining organic contaminations.

Ferritin from equine spleen (Sigma-Aldrich Chemie GmbH, Taufkirchen, Germany) was prepared at concentrations of 10 mg/mL and 30 mg/mL in PBS (pH 7.4, Sigma-Aldrich Chemie GmbH, Taufkirchen, Germany). Furthermore, 200 µL of the prepared protein solution was carefully placed on each substrate surface and incubated for 5 h at room temperature in an environmental chamber. After incubation, the substrates were rinsed with 1 mL of HPLC-grade water and dried in a stream of argon.

### 4.3. Atomic Force Microscopy (AFM)

The surface topographies of the Al_2_O_3_ substrates before and after ferritin adsorption were imaged in air using a Bruker Dimension ICON (Bruker France S.A.S., Wissembourg, France) in ScanAsyst Peak-Force Tapping mode with ScanAsyst-Air cantilevers (Bruker AFM Probes, Camarillo, CA, USA). The AFM images were analyzed with the Gwyddion open source software package (version 2.60) [44]. Additional AFM images are shown in Figures S1–S12.

### 4.4. X-ray Photoelectron Spectroscopy (XPS)

The chemical composition of the substrate surfaces before and after ferritin adsorption was analyzed using XPS. The measurements were carried out with an Omicron ESCA+ system (Omicron NanoTechnology, Taunusstein, Germany). A monochromatic Al Kα radiation (1486.7 eV) and a pressure below 4 × 10^−10^ mbar were applied. The samples were measured with a take-off angle of 30° with respect to the surface. The samples were neutralized with an emission current of 20 µA and a beam energy of 1 eV. For the survey and core level spectra, a pass energy of 100 eV and 20 eV was used, respectively. For the calibration, the Al2p peak at 74.1 eV was used, which bases on Al_2_O_3_. The background was subtracted with a Shirley type function, except for Fe2p, which was subtracted with a linear type function. The survey and high-resolution spectra of all samples are shown in Figures S13–S17.

### 4.5. Quantification and Statistical Analysis

The root-mean-square (RMS) surface roughness and the roughness factor were directly calculated for each image in Gwyddion. The pattern periodicity and amplitude, however, were determined from the 1D height–height correlation function calculated perpendicular to the ripples. In this function, the position of the first minimum corresponds to the pattern wavelength λ, while the square root of the intensity of the first maximum yields the peak-to-peak amplitude *A*_p2p_ [22]. All parameter values were averaged using OriginPro 2023 (OriginLab Corporation, Northampton, MA, USA) over five AFM images recorded at different positions on the substrate surfaces. The errors in Table 1 indicate the standard deviations.

The XP spectra were evaluated with CasaXPS V 2.3 (Casa Software Ltd., Teignmouth, UK) software.

## Figures and Tables

**Table 1:** Morphological parameters of the different Al_2_O_3_ substrate surfaces determined from the AFM images. Values represent averages of five AFM images recorded at different positions on the substrate surfaces with the standard deviations as errors.

	Flat	1300 °C	1400 °C	1500 °C
*S*_q_ (nm)	0.30 ± 0.02	8.43 ± 0.91	10.09 ± 0.26	7.98 ± 0.66
*r*	1.01 ± 0.01	1.08 ± 0.01	1.09 ± 0.01	1.09 ± 0.01
λ (nm)	-	162.3 ± 7.1	177.5 ± 13.2	145.9 ± 15.7
*A*_p2p_ (nm)	-	15.3 ± 1.5	18.4 ± 0.3	14.7 ± 1.1
*A*_p2p_/λ	-	0.094 ± 0.014	0.104 ± 0.009	0.100 ± 0.018

**Table 2:** Surface composition of the different Al_2_O_3_ substrate surfaces as determined by XPS. Corresponding survey spectra are shown in Figure S13.

	Flat	1300 °C	1400 °C	1500 °C
C 1s (at%)	8.0	16.1	12.9	10.7
O 1s (at%)	53.5	48.1	49.5	51.5
Al 2p (at%)	38.5	35.8	37.6	37.8
O 1s/Al 2p	1.39	1.34	1.32	1.36

## Data Availability

The data presented in this study are available on request from the corresponding author.

## Supplementary Materials

The supporting information can be downloaded at https://www.mdpi.com/article/10.3390/ijms241612808/s1.
