# Supplementary material for: Adsorption of Ferritin at Nanofaceted Al2O3 Surfaces
Source: Int J Mol Sci. 2023 Aug 15;24(16):12808. doi: 10.3390/ijms241612808 (PMC10454126; doi:10.3390/ijms241612808)
Supplement: Supplementary file 1 [file ijms-24-12808-s001.zip › ijms-2507835-supplementary.pdf]

Supplementary Materials

Adsorption of Ferritin at Nanofaceted Al_2O_3 Surfaces

Bhanu K. Pothineni [1,†], Sabrina Kollmann [1,†], Xinyang Li [1], Guido Grundmeier [1], Denise J. Erb [2,], and Adrian Keller [1,*]*

[1] Technical and Macromolecular Chemistry, Paderborn University, Warburger Str. 100, 33098 Paderborn, Germany

[2] Ion Beam Center, Institute of Ion Beam Physics and Materials Research, Helmholtz-Zentrum Dresden-Rossendorf, 01328 Dresden, Germany

[†] These authors contributed equally.

* Correspondence: d.erb@hzdr.de (D.J.E,); adrian.keller@uni-paderborn.de (A.K.); Tel.: +49-351-260-2898 (D.J.E.); Tel.: +49-5251-60-5722 (A.K.)

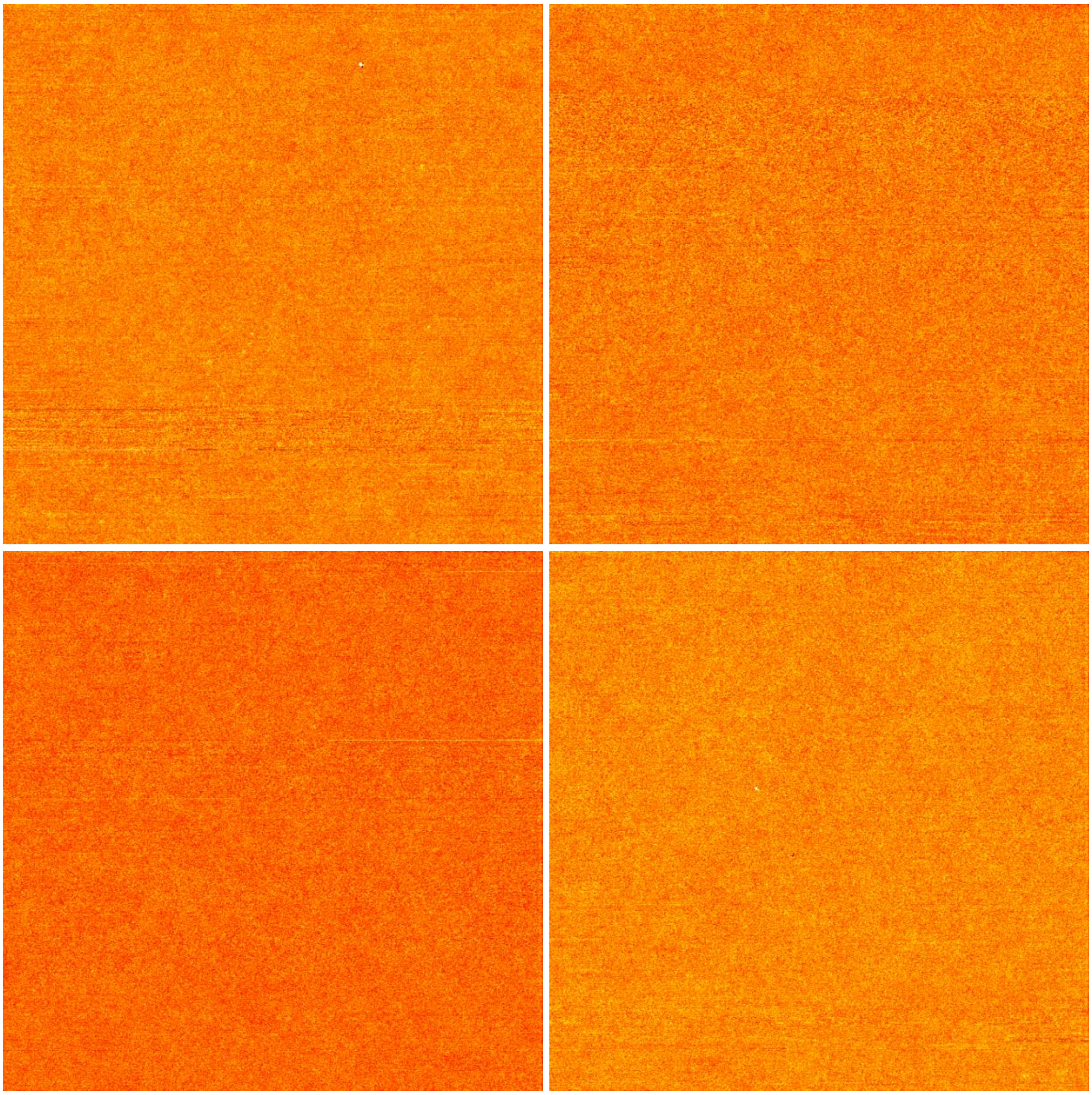

Figure S1: *Additional AFM topography images of flat* Al_2O_3 *substrates before ferritin adsorption. The images have a size of 3 x 3* μm^2 *and the maximum of the height scales is 4 nm.*

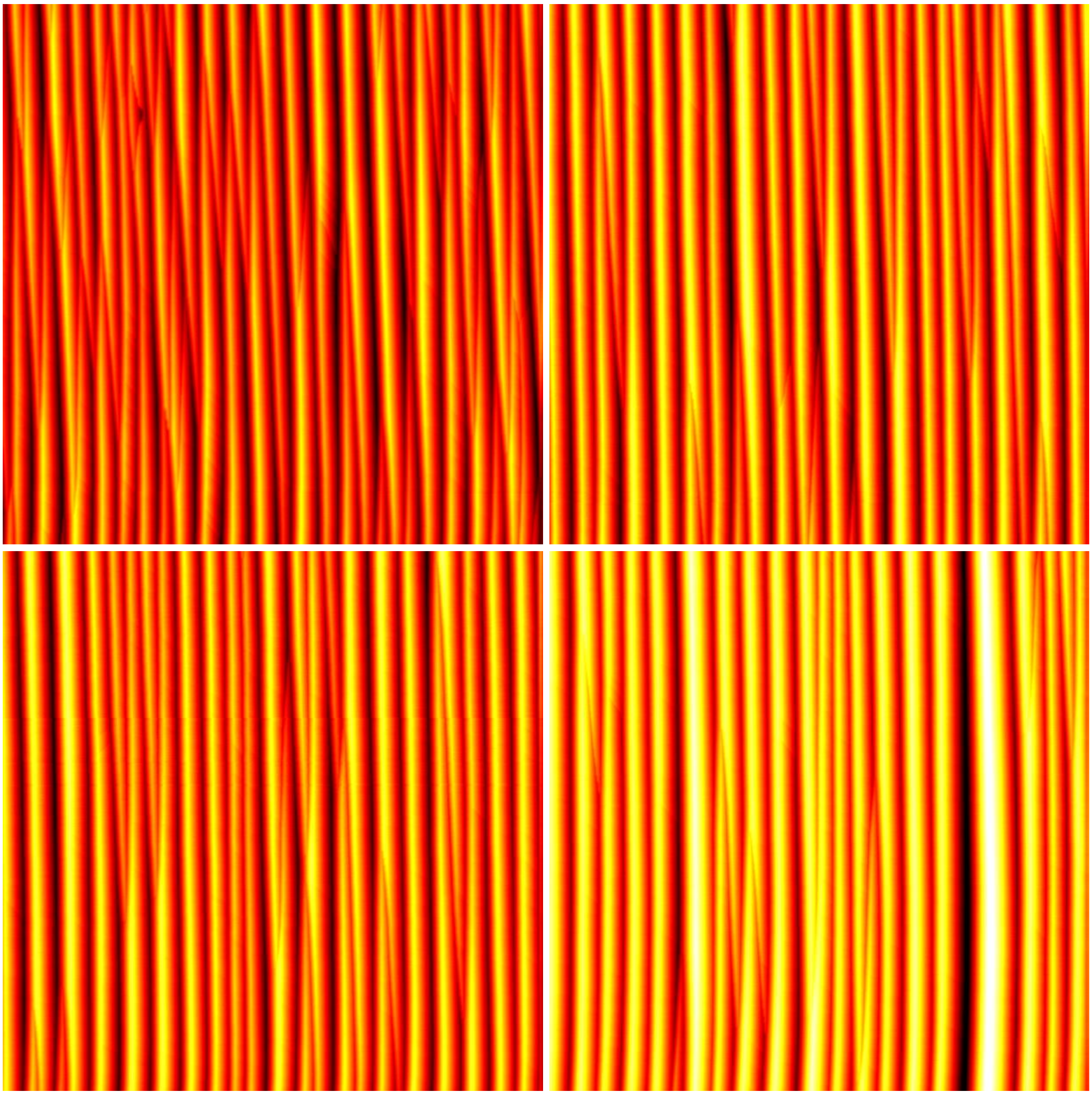

Figure S2: *Additional AFM topography images of nanofaceted* Al_2O_3 *substrates annealed at 1300 °C before ferritin adsorption. The images have a size of 3 x 3 µm² and the maximum of the height scales is 50 nm.*

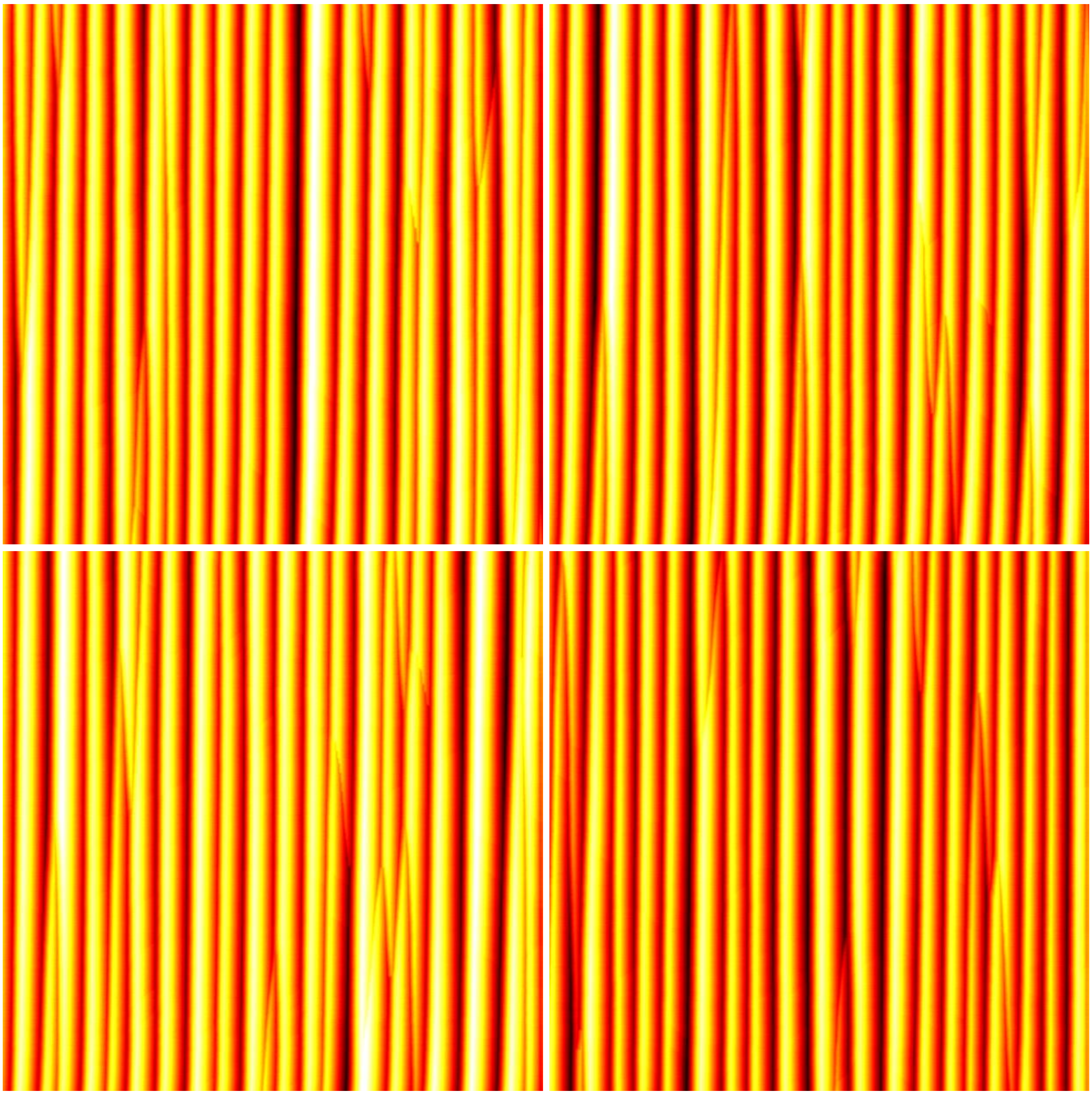

Figure S3: *Additional AFM topography images of nanofaceted Al_2O_3 substrates annealed at 1400 °C before ferritin adsorption. The images have a size of 3 x 3 µm² and the maximum of the height scales is 50 nm.*

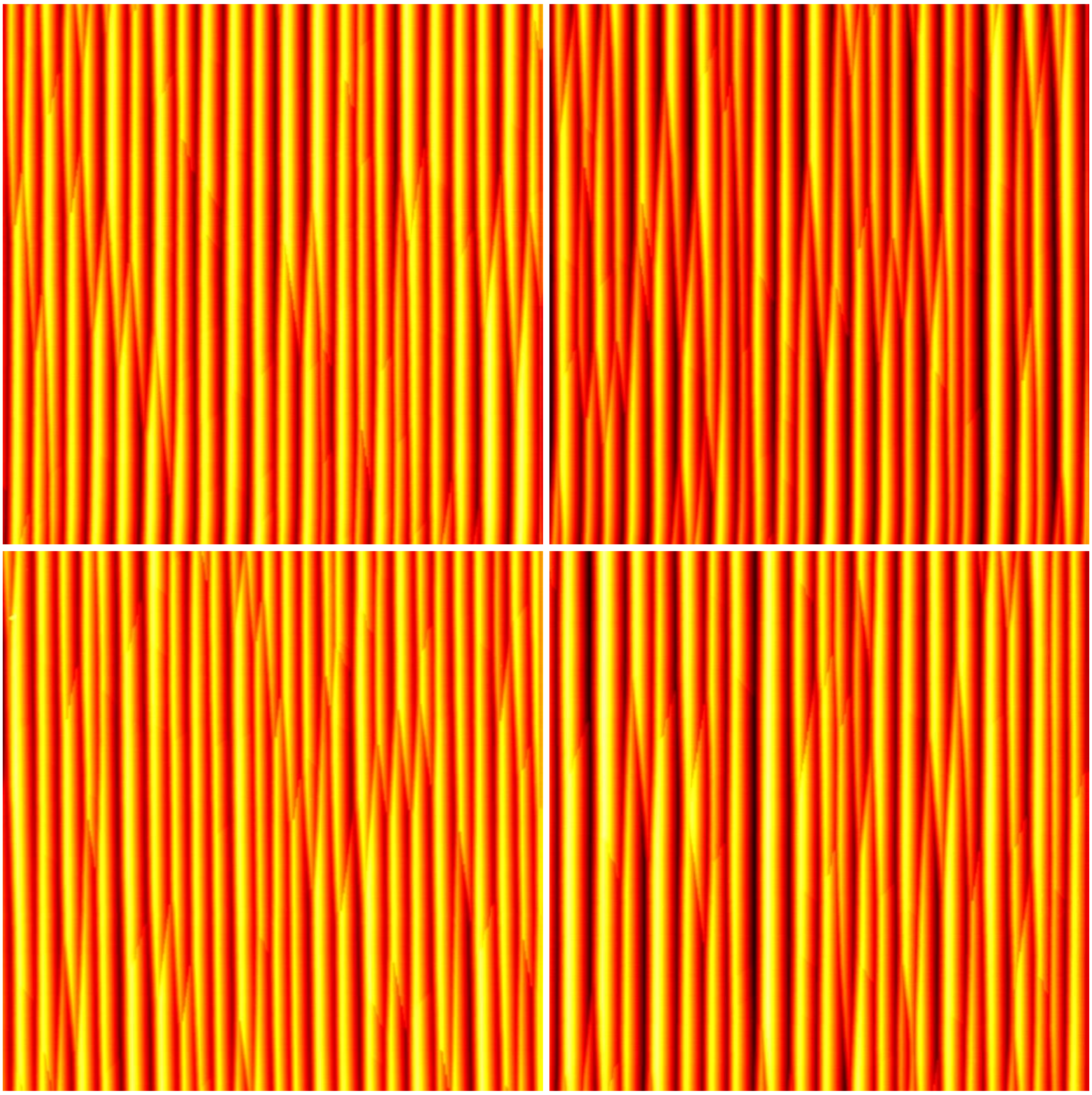

Figure S4: *Additional AFM topography images of nanofaceted* Al_2O_3 *substrates annealed at 1500 °C before ferritin adsorption. The images have a size of 3 x 3 µm² and the maximum of the height scales is 50 nm.*

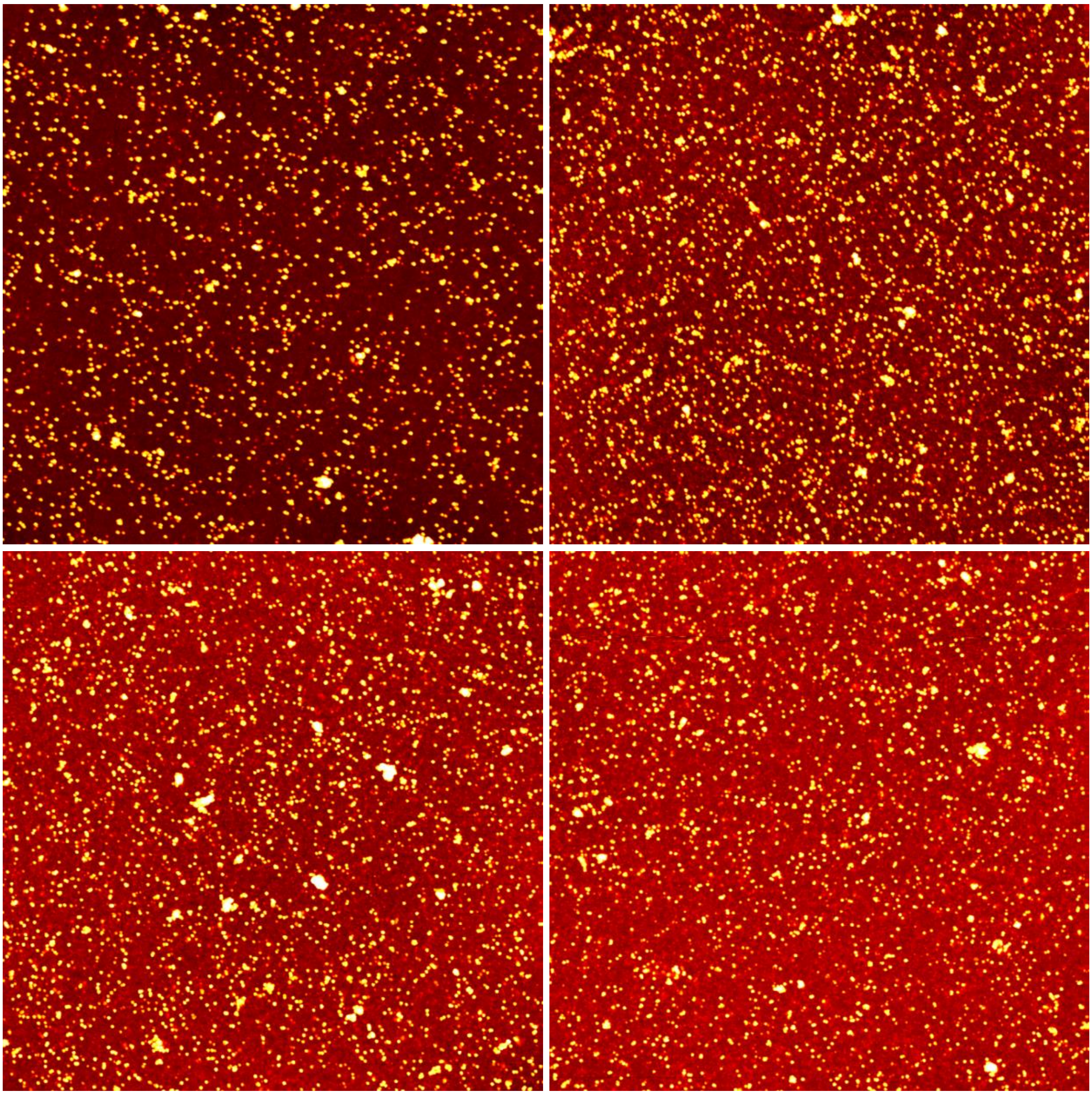

Figure S5: *Additional AFM topography images of flat Al_2O_3 substrates after ferritin adsorption at 10 mg/ml. The images have a size of 3 x 3 µm² and the maximum of the height scales is 12 nm.*

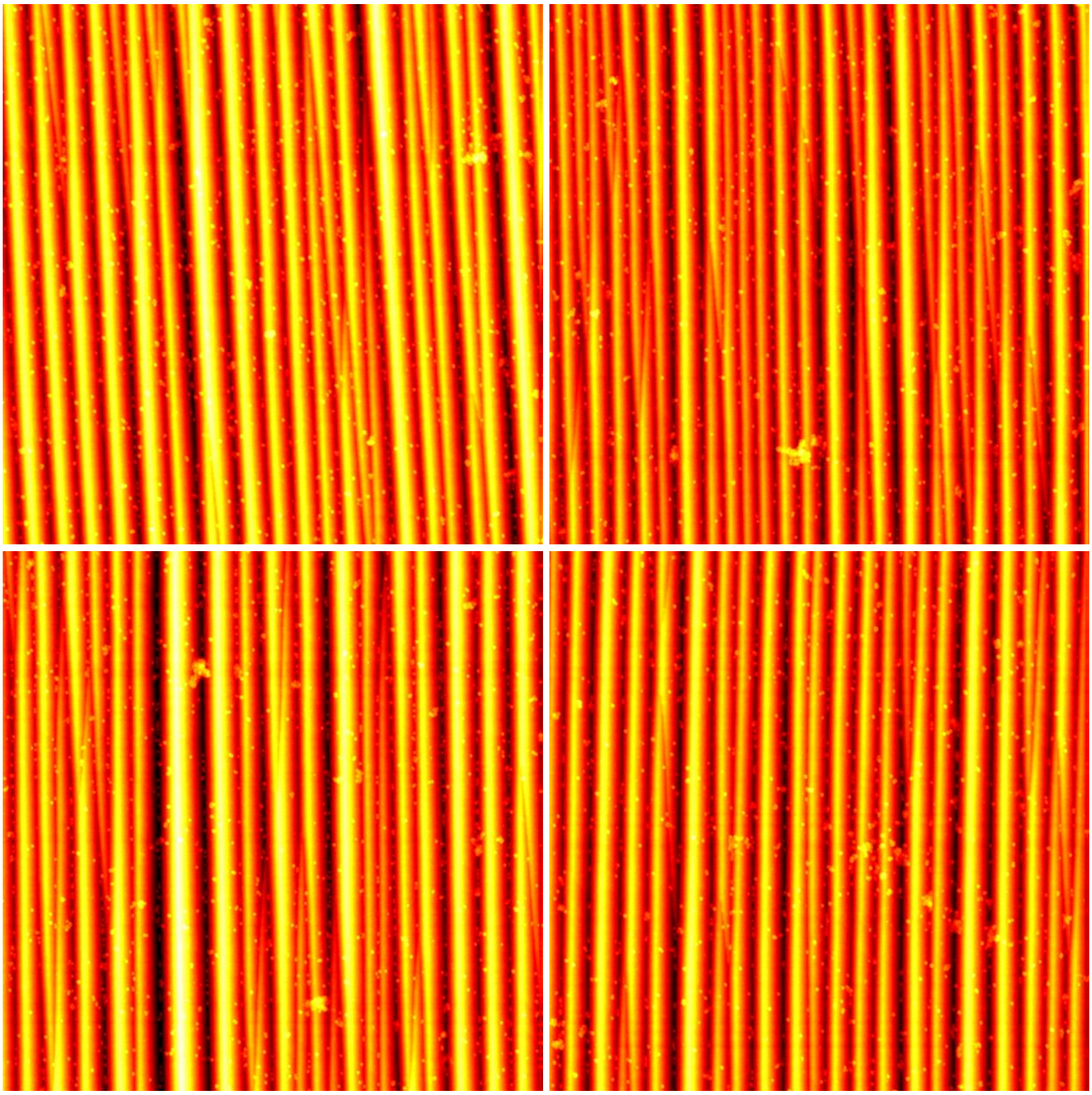

Figure S6: *Additional AFM topography images of nanofaceted* Al_2O_3 *substrates annealed at 1300 °C after ferritin adsorption at 10 mg/ml. The images have a size of 3 x 3 µm² and the maximum of the height scales is 50 nm.*

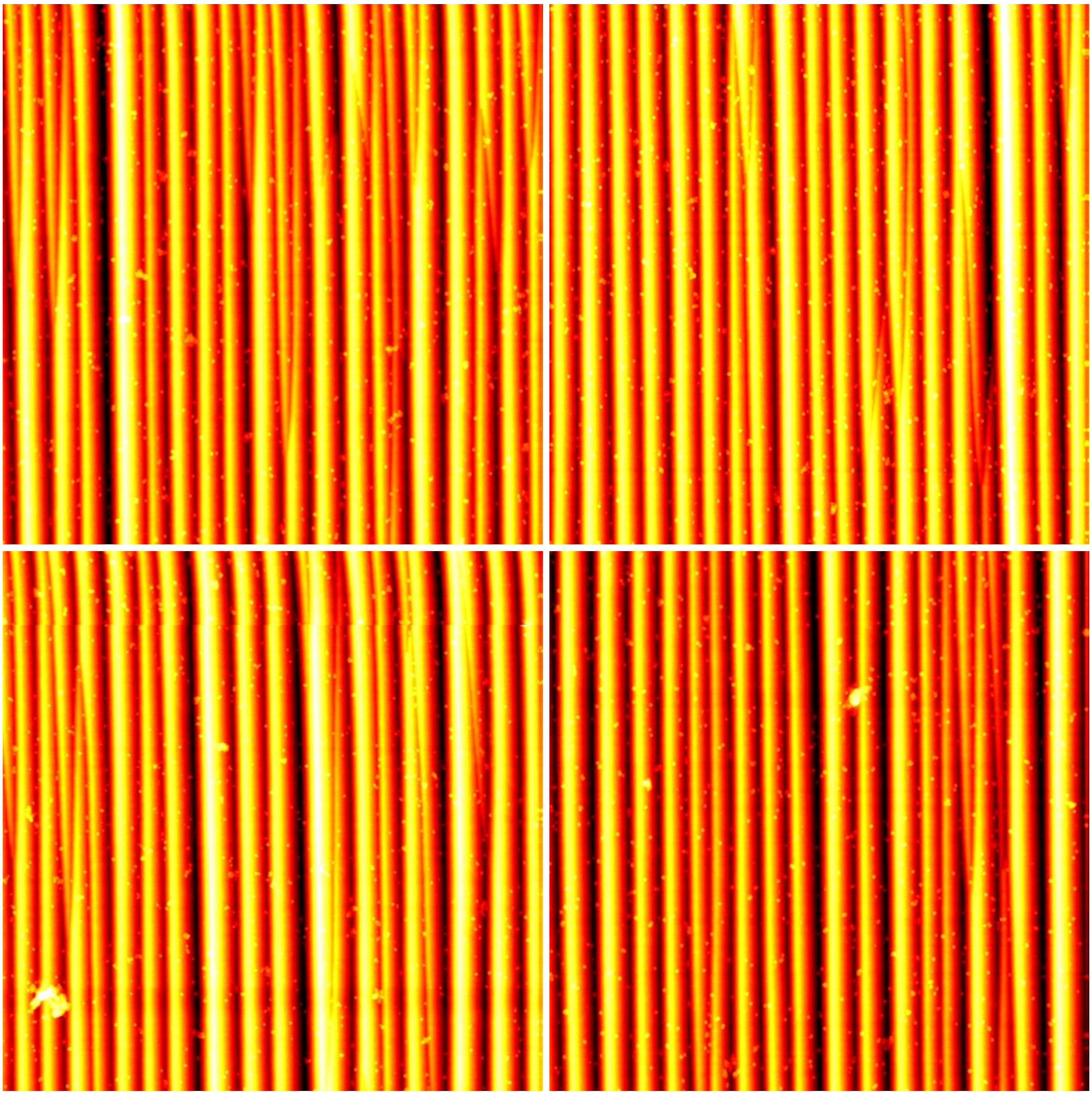

Figure S7: *Additional AFM topography images of nanofaceted Al_2O_3 substrates annealed at 1400 °C after ferritin adsorption at 10 mg/ml. The images have a size of 3 x 3 µm² and the maximum of the height scales is 50 nm.*

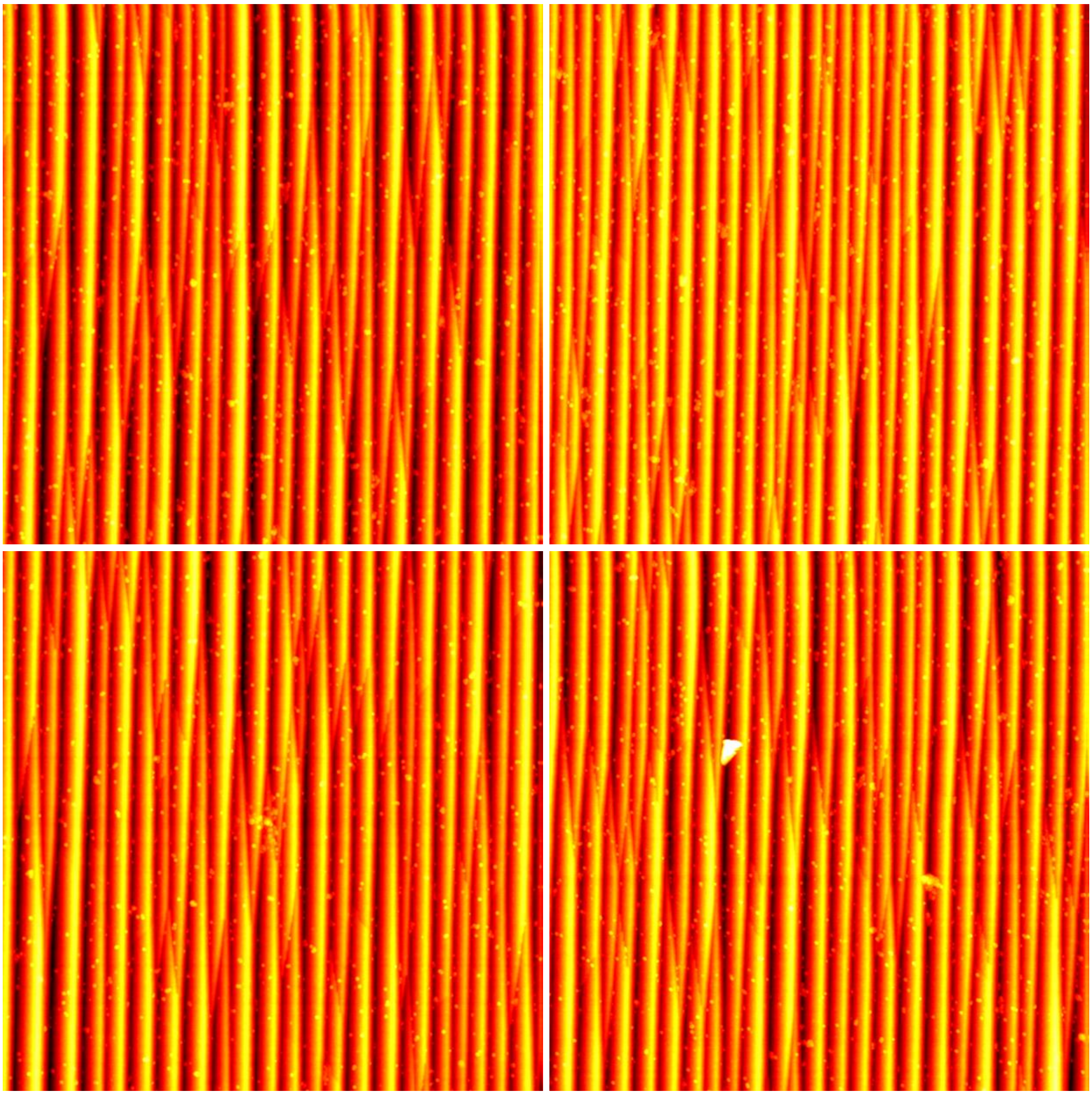

Figure S8: *Additional AFM topography images of nanofaceted* Al_2O_3 *substrates annealed at 1500 °C after ferritin adsorption at 10 mg/ml. The images have a size of 3 x 3 µm² and the maximum of the height scales is 50 nm.*

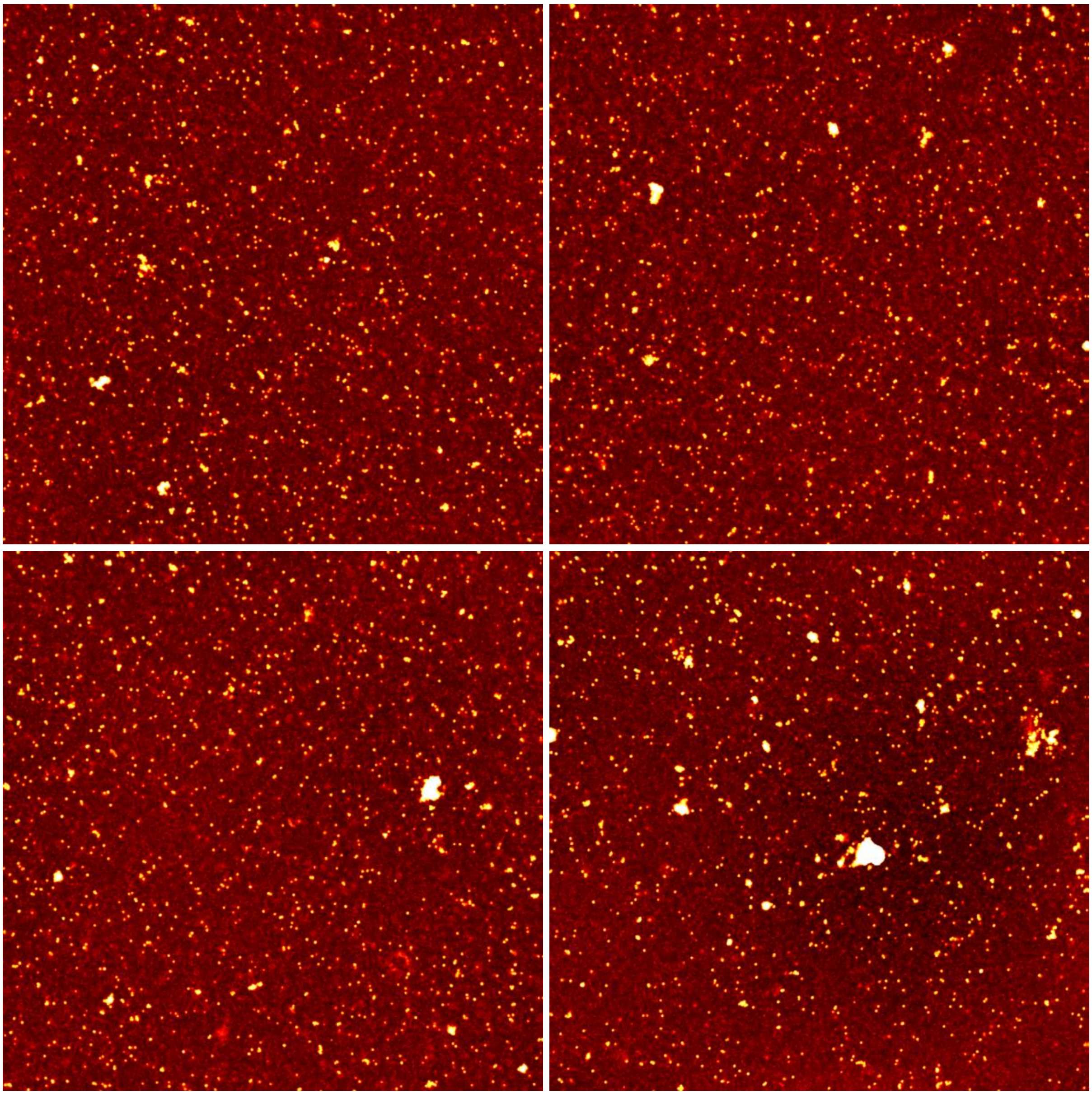

Figure S9: *Additional AFM topography images of flat Al_2O_3 substrates after ferritin adsorption at 30 mg/ml. The images have a size of 3 x 3 µm² and the maximum of the height scales is 12 nm.*

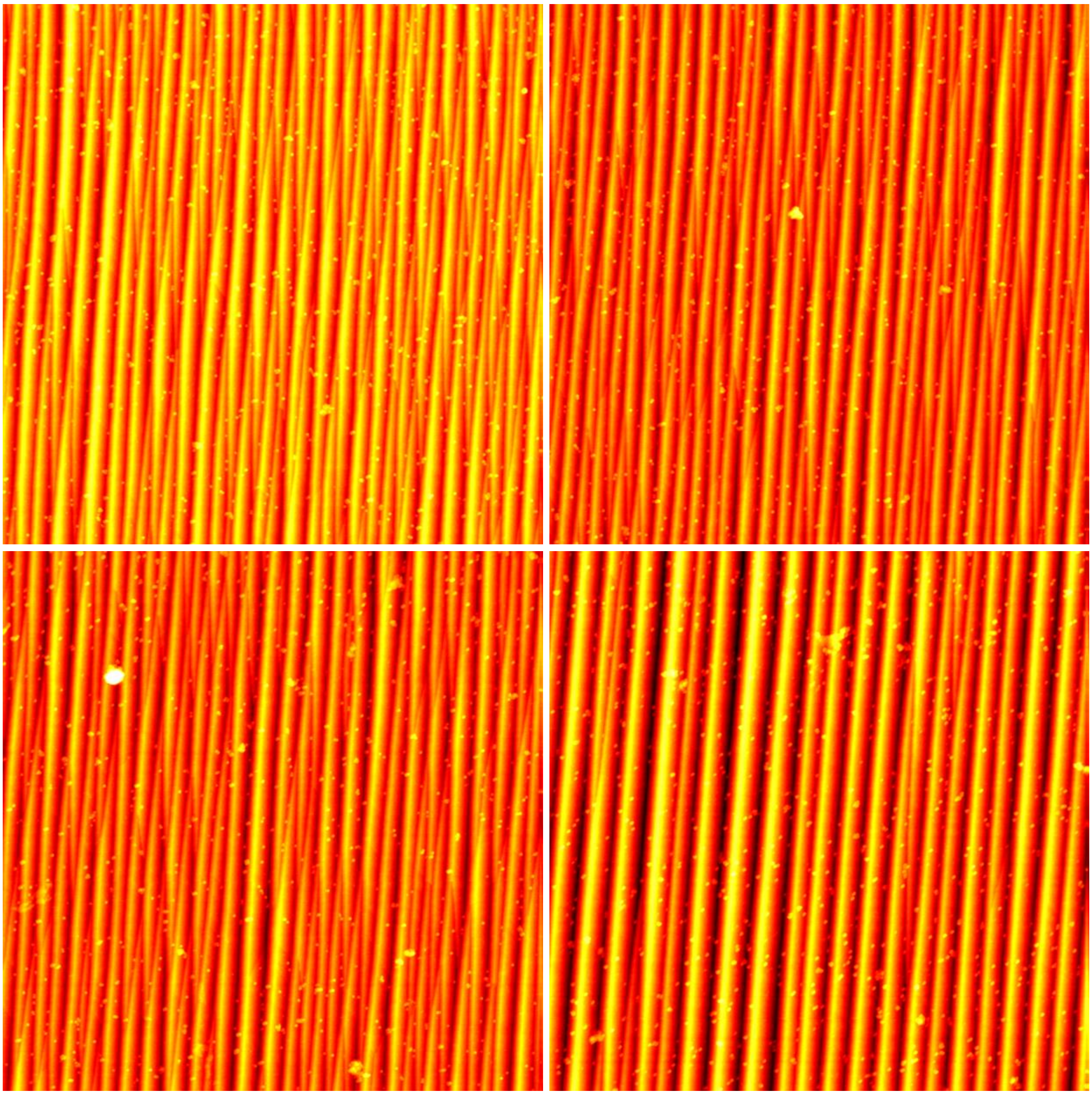

Figure S10: *Additional AFM topography images of nanofaceted* Al_2O_3 *substrates annealed at 1300 °C after ferritin adsorption at 30 mg/ml. The images have a size of 3 x 3 µm² and the maximum of the height scales is 50 nm.*

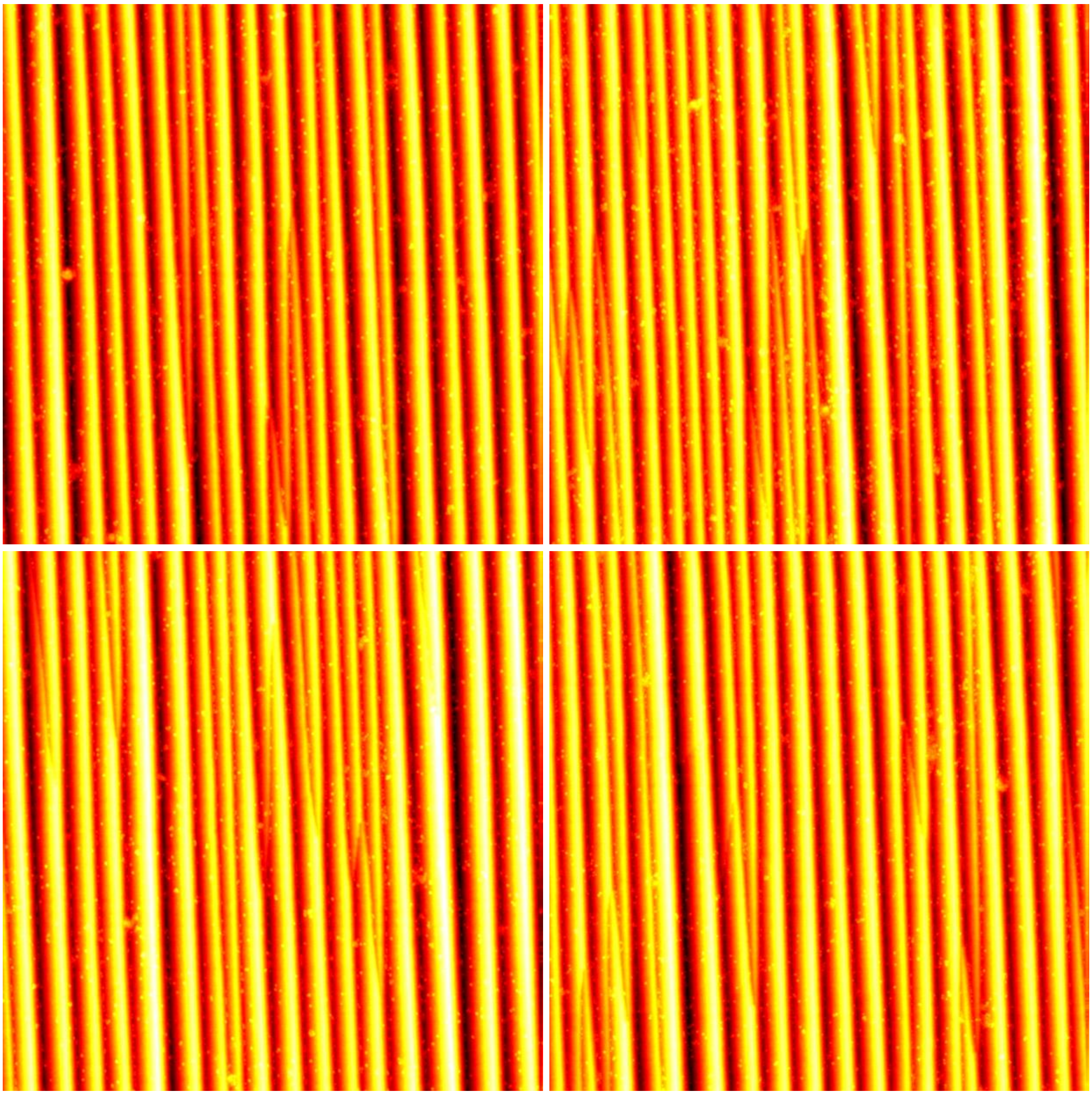

Figure S11: *Additional AFM topography images of nanofaceted* Al_2O_3 *substrates annealed at 1400 °C after ferritin adsorption at 30 mg/ml. The images have a size of 3 x 3 µm² and the maximum of the height scales is 50 nm.*

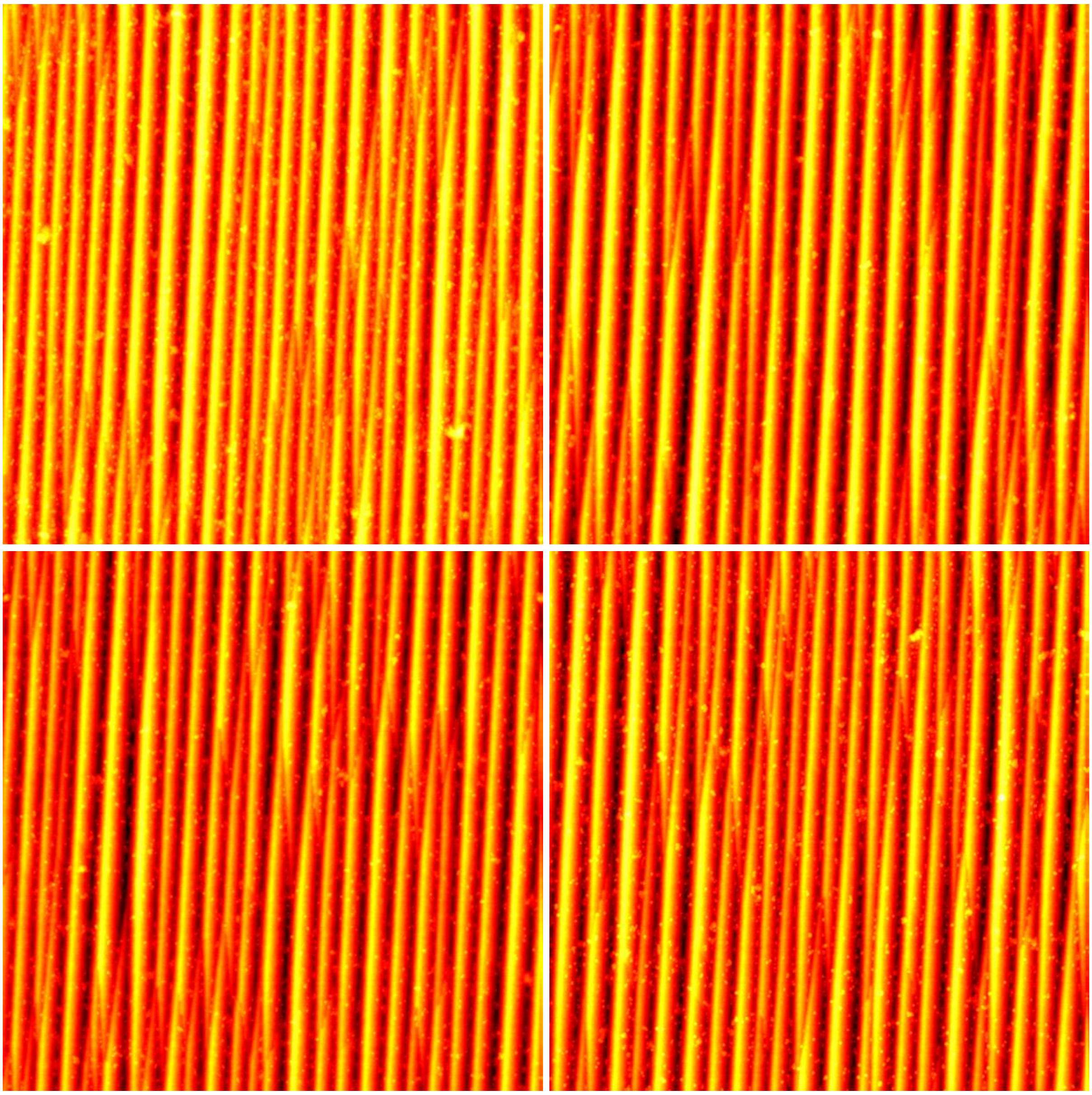

Figure S12: *Additional AFM topography images of nanofaceted* Al_2O_3 *substrates annealed at 1500 °C after ferritin adsorption at 30 mg/ml. The images have a size of 3 x 3 µm² and the maximum of the height scales is 50 nm.*

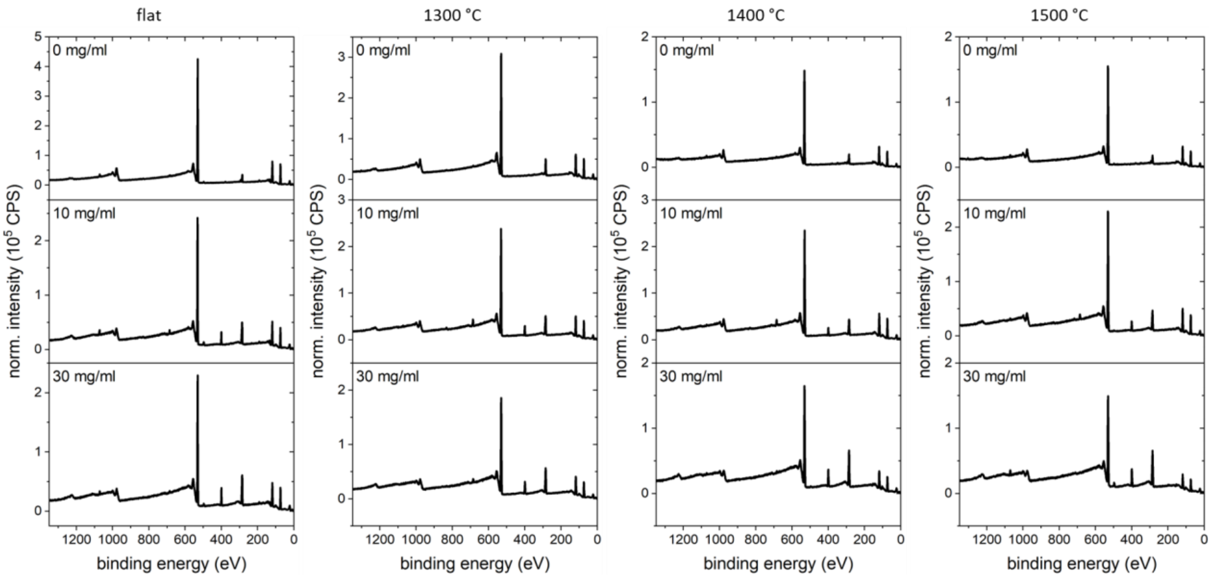

Figure S13: *Survey spectra of the four* Al_2O_3 *substrate surfaces before and after ferritin adsorption at 10 and 30 mg/ml, respectively.*

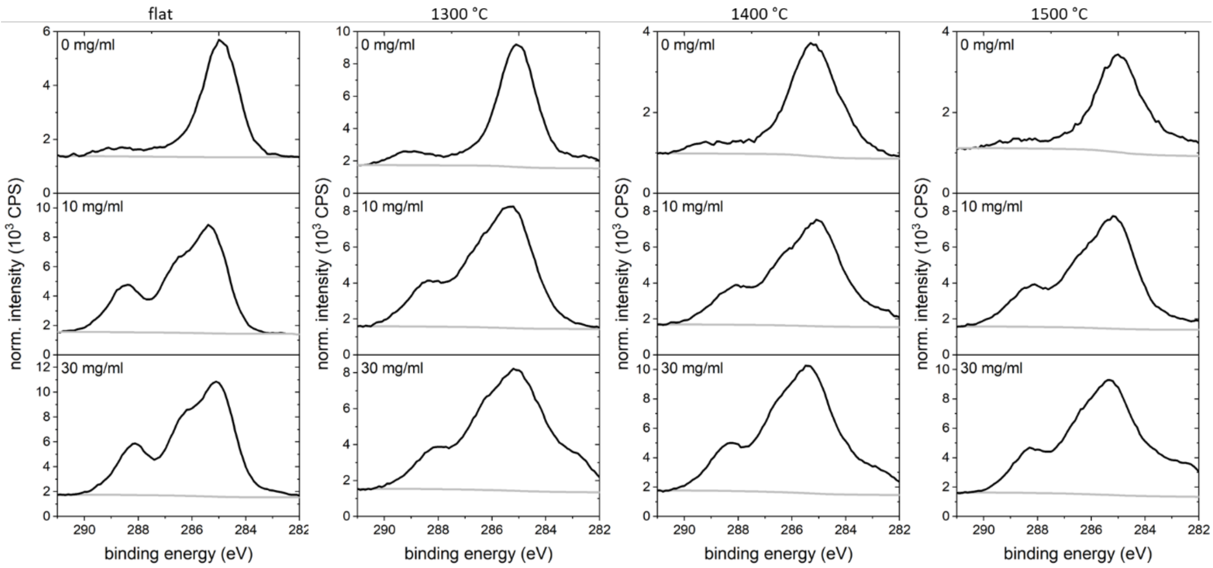

Figure S14: *High-resolution C 1s spectra of the four* Al_2O_3 *substrate surfaces before and after ferritin adsorption at 10 and 30 mg/ml, respectively. The background used for quantification is indicated in grey.*

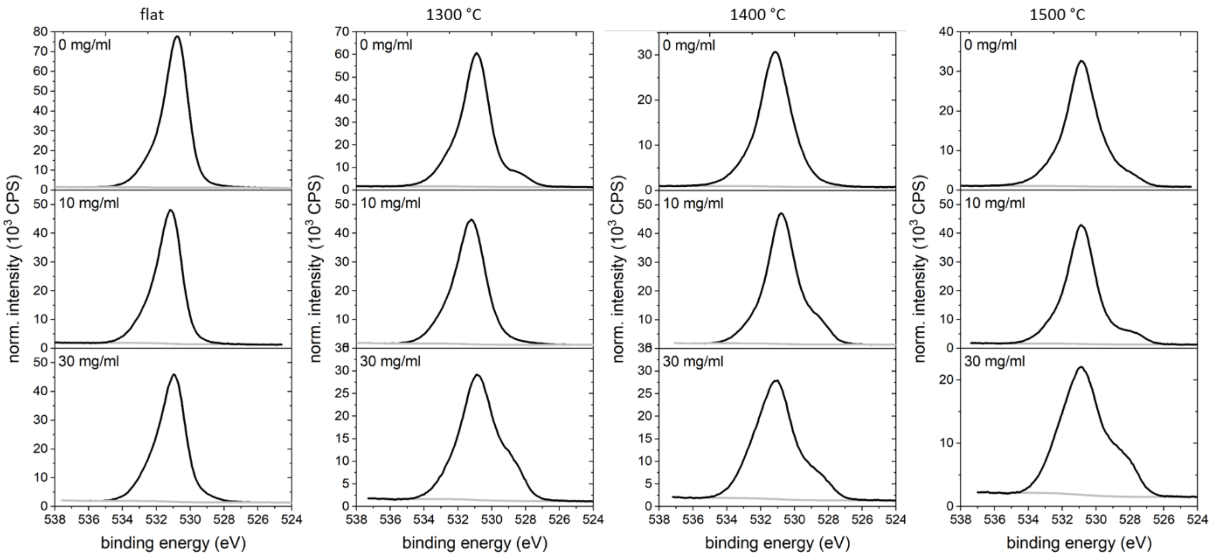

Figure S15: *High-resolution O 1s spectra of the four Al_2O_3 substrate surfaces before and after ferritin adsorption at 10 and 30 mg/ml, respectively. The background used for quantification is indicated in grey.*

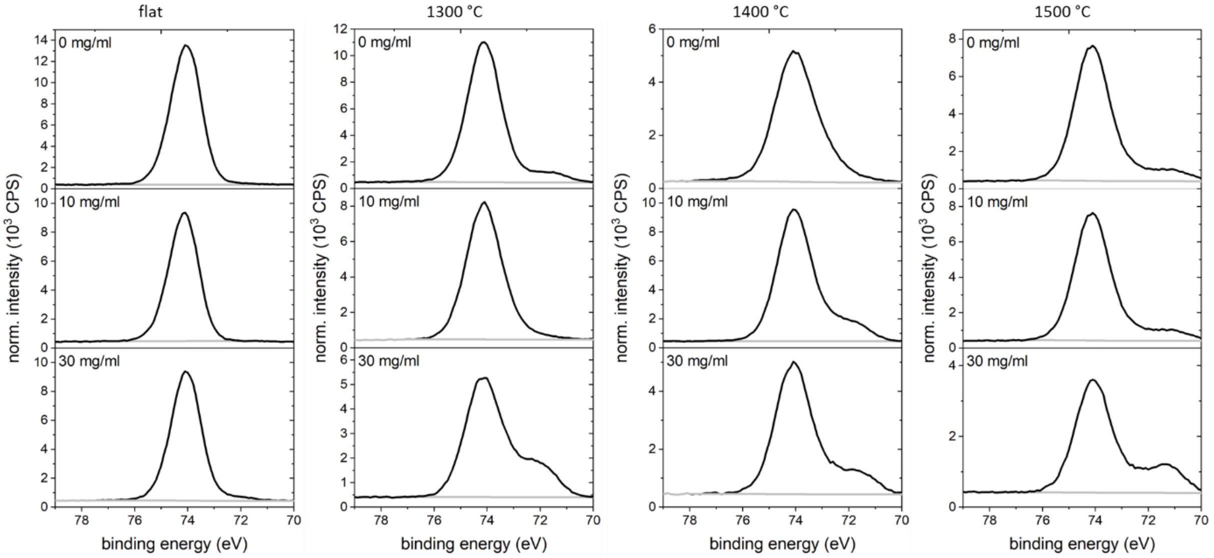

Figure S16: *High-resolution Al 2p spectra of the four Al_2O_3 substrate surfaces before and after ferritin adsorption at 10 and 30 mg/ml, respectively. The background used for quantification is indicated in grey.*

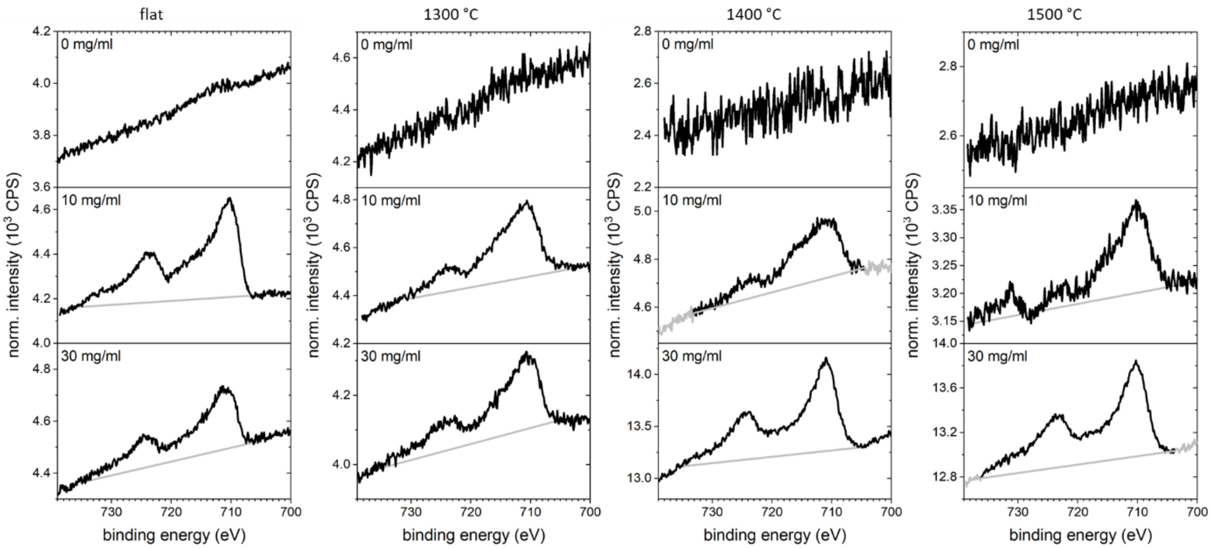

Figure S17: *High-resolution Fe 2p spectra of the four* Al_2O_3 *substrate surfaces before and after ferritin adsorption at 10 and 30 mg/ml, respectively. The background used for quantification is indicated in grey.*
